# Optical Coherence Tomography Angiography–Based Evaluation of Foveal Avascular Zone and Macular Vessel Density in Prediabetic Patients

**DOI:** 10.5152/eurasianjmed.2026.251165

**Published:** 2026-01-10

**Authors:** Bahadır Utlu, Elif Sedanur Utlu, Emine Çinici, Hasan Akgöz, Kemal Bayrakçeken, Betül Dertsiz Kozan

**Affiliations:** 1 Department of Ophthalmology , Erzurum Regional Training and Research Hospital, Erzurum, Türkiye; 2 Department of Family Medicine, Erzurum Regional Training and Research Hospital, University of Health Sciences, Erzurum, Türkiye; 3 Department of Ophthalmology, Atatürk University Faculty of Medicine, Erzurum, Türkiye; 4 Department of Ophthalmology, Erzincan Binali Yıldırım University Faculty of Medicine, Erzincan, Türkiye; 5 Department of Ophthalmology, Gazi Yaşargil Training and Research Hospital, Diyarbakır, Türkiye

**Keywords:** Macula lutea, microcirculation, optical coherence tomography, prediabetic state, retina, retinal vessels

## Abstract

**Background:**

To assess macular layer thickness, macular vessel density, and foveal avascular zone (FAZ) parameters in prediabetic patients compared with healthy normoglycemic controls using optical coherence tomography angiography (OCTA).

**Methods:**

Thirty prediabetic patients (group A; fasting plasma glucose 100-125 mg/dL, postprandial plasma glucose 140-199 mg/dL, glycated hemoglobin 5.7%-6.4%) and 30 age-matched normoglycemic subjects (group B) were included. The OCTA imaging was used to evaluate superficial capillary plexus (SCP) and deep capillary plexus (DCP) vessel densities, as well as FAZ area and perimeter. Participants with poor image quality, high refractive error, glaucoma, prior intraocular surgery, chorioretinal atrophy, or other ocular/systemic comorbidities were excluded.

**Results:**

TheSCP and DCP perfusion densities (PDs) were lower in the prediabetic group, with significant reductions in the inferior and temporal quadrants of the DCP and the temporal quadrant of the SCP (*P* < .05). The FAZ area and perimeter were larger in the prediabetic group but not statistically significant (*P* > .05). Macular thickness was greater in all quadrants in group A, with significant thickening in the nasal and inferior quadrants (*P* < .05).

**Conclusion:**

Prediabetic patients demonstrated early microvascular and structural changes, including reduced macular PD, increased macular thickness, and FAZ enlargement. These findings suggest subclinical retinal involvement in prediabetes, warranting larger longitudinal studies.

**Main Points**

- Even before clinical signs of retinopathy appear, a reduction in macular vessel density can be detected in prediabetic individuals using optical coherence tomography angiography (OCTA).
- The deep capillary plexus appears to be the most vulnerable region to early microvascular damage during the prediabetic stage.
- Increased thickness in the nasal and inferior macular regions was observed in the prediabetic group, which may indicate early metabolic stress and subclinical edema.
- The foveal avascular zone showed a slight tendency toward enlargement, although these differences were not statistically significant.
- These findings suggest that OCTA may serve as a valuable screening tool for detecting subclinical microvascular changes in prediabetic individuals.

## Introduction

Prediabetes is widely recognized as an intermediate metabolic state that lies on the spectrum between normal glucose regulation and overt diabetes mellitus. In this condition, circulating glucose concentrations rise above physiologic norms but do not yet meet the internationally accepted diagnostic criteria for diabetes.^1^ Rather than being a stable or harmless phase, prediabetes is increasingly regarded as a dynamic stage in the natural history of diabetes, during which various metabolic disturbances begin to emerge. According to projections by the International Diabetes Federation, approximately 470 million adults across the globe are expected to exhibit impaired glucose regulation by 2030, which would represent about 6% of the world’s adult population.^2^ Longitudinal epidemiological studies have consistently shown that between 5% and 10% of individuals with prediabetes transition to overt diabetes each year, especially when risk factors such as central obesity, physical inactivity, dyslipidemia, or hypertension are present. ^3^^,^^4^ This progression is not inevitable, but it underscores the critical need to identify early markers of microvascular dysfunction that may predict future disease development.

Beyond its role as a precursor to diabetes, prediabetes has significant clinical implications because it is already associated with an increased burden of both macrovascular and microvascular complications. A growing body of evidence has linked prediabetes to a higher incidence of cardiovascular disease, chronic kidney disease, peripheral neuropathy, and diabetic retinopathy, even before the appearance of persistent hyperglycemia.^5^ This suggests that vascular injury may begin much earlier than previously assumed. The underlying mechanism is thought to involve chronic low-grade hyperglycemia, which induces oxidative stress, promotes endothelial dysfunction, and disrupts the autoregulation of blood flow. These processes can lead to microvascular remodeling and compromise tissue perfusion. The retina, with its exceptionally high oxygen consumption and dense microvascular network, is particularly vulnerable to these early metabolic and hemodynamic disturbances.^6^

In recent years, optical coherence tomography angiography (OCTA) has emerged as a powerful, noninvasive imaging modality capable of providing high-resolution, depth-resolved visualization of the retinal and choroidal microvasculature. Unlike conventional fluorescein angiography, which requires intravenous dye injection and primarily reveals areas of leakage or gross nonperfusion, OCTA enables the quantitative assessment of capillary-level flow without the need for contrast agents. It can measure vessel density in the superficial capillary plexus (SCP) and deep capillary plexus (DCP), map the size and shape of the foveal avascular zone (FAZ), and evaluate central macular thickness (CMT) and other structural parameters.^7^^,^^8^ Because these measurements can be obtained rapidly and noninvasively, OCTA offers an attractive approach for detecting subtle retinal microvascular changes at a stage when no clinical signs of diabetic retinopathy are yet visible.^9^

Several studies have reported that individuals with prediabetes exhibit early alterations in retinal microvasculature, such as reduced parafoveal vessel density, enlargement or shape irregularities of the FAZ, and thinning of the ganglion cell complex (GCC).^10^ These changes are often mild and localized, which may explain the variability in findings across studies and the lack of consensus in the literature. Nonetheless, such subclinical abnormalities may represent the earliest manifestations of diabetic microangiopathy. Identifying them could have substantial clinical significance, allowing for timely lifestyle or pharmacological interventions aimed at halting or slowing the transition from prediabetes to diabetes.

Given the increasing global burden of prediabetes and its potential for causing silent microvascular damage, there is a pressing need to investigate objective, reproducible imaging biomarkers that can be used in routine clinical practice. In this context, OCTA provides a unique opportunity to study the retinal vasculature in vivo with high precision. The present study was therefore designed to comprehensively evaluate retinal microvascular and structural changes in individuals with prediabetes using OCTA, with the ultimate aim of identifying early biomarkers of microangiopathy. By clarifying the nature and pattern of these early changes, this work may contribute to the development of more effective strategies for early detection, risk stratification, and prevention of diabetes-related complications.

## Material and Methods

The study, which was planned to be conducted using a cross-sectional observational methodology, was completed in a period of approximately 6 months. Prediabetes was defined as individuals with blood glucose levels meeting at least one of the criteria specified in the introduction, according to the ADA (American Diabetes Association) criteria.^1^ This approach was chosen to reflect the heterogeneity of the prediabetic population, consistent with previous studies.^3^^,^^4^ Individuals with any ocular or systemic disease that could affect the study outcomes were excluded from the study. In this context, ocular pathologies that may influence the structure of the retina and macula or alter retinal microcirculation (such as glaucoma, retinal or choroidal diseases, and vitreous or corneal pathologies), as well as systemic conditions other than diabetes that could affect vascular integrity (including hypertension, cardiovascular diseases, chronic kidney disease, and systemic inflammatory disorders), were among the exclusion criteria. Those who had undergone previous eye surgery, those with glaucoma or vitreous or corneal pathology, and those with various diseases and conditions likely to affect the cardiovascular system were excluded from the study. Although an attempt was made to exclude participants with neurological disorders that could affect the retinal vasculature (e.g., Alzheimer’s disease), this was considered a study limitation due to the difficulty of anamnesis and diagnostic difficulties.^6^^,^^11^

30 healthy individuals (group B) who did not have systemic diseases, were non-smokers, and had no ocular disease were included as controls. These individuals were generally emmetropic individuals without any refractive error and were selected from among those applying to the outpatient clinic for administrative tasks such as starting work. The study was approved by the University of Health Sciences Erzurum Medical Faculty Ethics Committee, dated May 12, 2021, numbered 2021/254-36. All participants were included in the study after written informed consent was obtained. The entire study was conducted in accordance with the ethical standards of the Declaration of Helsinki.

All participants underwent a comprehensive medical and ophthalmological evaluation. Blood glucose parameters (fasting plasma glucose, 2-hour postprandial plasma glucose, and glycated hemoglobin (HbA1c)) were measured to confirm compliance with ADA criteria. Visual acuity, biomicroscopic examination, and anterior segment examinations were performed. Intraocular pressure was recorded using a tonometer. After inducing pharmacological mydriasis, a fundus examination was performed, and color fundus photographs were obtained using a Carl Zeiss Visucam Pro Fundus Camera® (Carl Zeiss Meditec AG, Jena, Germany). Where appropriate, the presence and stage of non-proliferative diabetic retinopathy (NPDR) were documented.

The OCTA scans were acquired with the RS-3000 Advance 2 device (Nidek®), which includes the AngioScan software version 2.0 for image analysis. All scans were obtained by a single experienced ophthalmologist at the same time of day under standard room illumination (~40 lux) to minimize diurnal variation.^7^^,^^8^^,^^12^ The technical parameters of the device used included 20 micrometer transverse resolution, 53 000 scans per second A-scan speed, 7 micrometer axial resolution, and a light source centered at 880 nm. A 3 × 3 mm and 6 × 6 mm macular cube scans containing 256 B-scans (6 × 12 lines) centered at the fovea were obtained using internal fixation.^12^ Retinal layers were segmented automatically as follows: the SCP from the inner limiting membrane to 30 micrometer below the inner plexiform layer (IPL), the DCP from 15 micrometer to 70 micrometer below the IPL, and the outer retina plus choriocapillaris from 70 micrometer below the IPL to 30 micrometer beneath the retinal pigment epithelium.^9^^,13^ Manual corrections were made when needed. The evaluated parameters included vessel density in the SCP and DCP, their thicknesses, FAZ area, perimeter, circularity, and CMT. Only scans with a signal strength index of ≥5 were included.

Data analysis was performed using SPSS software, version 21.0. Quantitative variables were summarized as averages with their variability indicators and also as median values accompanied by the range encompassing the middle half of the data, whereas qualitative variables were described by their frequencies and proportions. The distribution characteristics of the data were first examined to determine whether they followed a normal pattern. For group comparisons, relationships between categorical variables were explored with tests designed to evaluate differences in proportions, while differences in numerical variables were assessed using either methods suitable for data with normal distribution or alternative rank-based methods when the distributional assumptions were not met. In all analyses, a probability value below 0.05 was accepted as the threshold for statistical significance.

## Results

**Figure 1.**
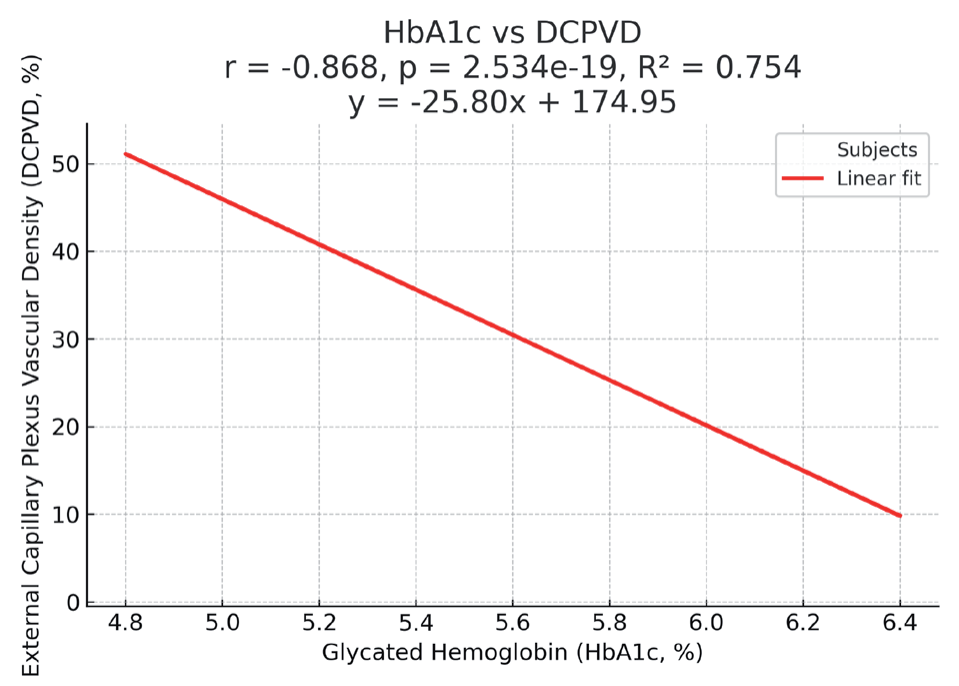
Scatter plot showing the relationship between glycated hemoglobin (HbA1c, %) and external capillary plexus vascular density (DCPVD, %). The red line represents the linear regression fit (*r* = −0.868, *P* < .001, *R*^2^ = 0.754). Higher HbA1c levels are associated with lower vascular density.

A total of 60 eyes of 30 prediabetic and 30 healthy participants were included in the study. The mean age was 41.0 ± 2.3 years in the prediabetic group and 40.9 ± 3.0 years in the control group. Mean HbA1c was 6.1 ± 0.19% in the prediabetic group and 5.2 ± 0.2% in the control group. Fundus examination revealed no findings in all control patients, while 5 of the prediabetic patients exhibited minimal intraretinal hemorrhage and irregular macular reflexes and were classified as early NPDR. No abnormalities were observed in the control group. Table 1 summarizes the perfusion density (PD) values of the SCP and DCP (Table 1). Vessel density values were lower in both plexuses in the prediabetic group compared to the control group. These reductions reached statistical significance in the temporal quadrant of the SCP (*P* < .05) and in the inferior and temporal quadrants of the DCP (*P* < .05). Table 2 presents the FAZ parameters, including FAZ area, perimeter, and circularity (Table 2). Although FAZ area and perimeter were slightly larger, and FAZ circularity was lower in the prediabetic group compared to the control group, none of these differences were statistically significant (*P* > .05). Table 3 shows the macular and GCC thickness values. Prediabetic patients showed significantly greater thickness in the nasal and inferior zones of the outer retina and in the inferior zone of the inner retina (*P* < .05), but no difference was found in CMT (Table 3). The relationship between HbA1c levels and DCP vessel density is shown in Figure 1. The GCC thickness did not differ significantly between groups, but a trend toward higher values was seen in prediabetic patients.

## Discussion

The study revealed several early retinal alterations in individuals with prediabetes, even in the absence of clinically detectable diabetic retinopathy. Compared with healthy controls, prediabetic patients showed a general reduction in vessel density in both the superficial and DCPs, with these changes being more evident in specific quadrants rather than uniformly distributed across the macula. Although the FAZ tended to appear slightly enlarged and less circular in prediabetic subjects, these changes were subtle and not markedly distinct from those observed in healthy eyes, suggesting that FAZ metrics alone may not be sensitive indicators of very early disease. Structural analysis demonstrated localized thickening in certain macular subfields, while overall CMT and GCC thickness remained largely comparable between groups. Taken together, these findings indicate that retinal microvascular and structural abnormalities may begin to develop during the prediabetic stage in a patchy and subclinical manner, preceding overt diabetic retinopathy.

The OCTA technology allows for multiple, detailed images of retinal capillaries without invasive intervention. It allows quantitative assessment of vessel density and visualization of early microangiopathic changes in diabetes.^14^ The FFA has traditionally been labeled as the gold standard for evaluating capillary nonperfusion in proliferative diabetic retinopathy, but this view has been challenged, as the diagnosis of diabetic retinopathy largely relies on clinical examination rather than angiographic findings. Moreover, FFA carries systemic contraindications and side effects due to intravenous dye injection, which can cause patient discomfort.^15^ In contrast, OCTA is safe, repeatable, and has emerged as a valuable tool for detecting microvascular alterations before clinical signs of retinopathy appear.

Many studies have reported that vascular density decreases along with dysregulation of the FAZ in diabetic individuals.^16^^-^^18^ The OCTA vessel density algorithms have shown that SCP and DCP PD decreases chronically in eyes with diabetic retinopathy.^19^^-^^22^ However, findings are less consistent in the prediabetic phase. Previous studies have reported inconsistent findings, with some showing similar PDs between prediabetic patients and healthy controls, while others observed a decrease in SCP and DCP PD.^23^ The results appear consistent with the literature, demonstrating significantly lower PD in the temporal sector of the SCP and the inferior and temporal sectors of the DCP, with a non-significant decreasing trend in the remaining quadrants. These results suggest that microvascular changes in prediabetes may occur sporadically rather than uniformly affecting the entire macular region. Variability among previous studies may be due to differences in sample size, OCTA device characteristics, analysis software, and demographic factors such as age, gender, and ethnicity.

The FAZ is metabolically vulnerable due to its high oxygen demands.^24^Enlargement of FAZ area and reduction of circularity have been linked to progression of diabetic retinopathy.^25^^,^^26^ However, studies in prediabetes have yielded inconsistent results, with several showing no significant differences compared to controls.^24^^,^^27^ In this study, no statistically significant differences were found between groups in terms of FAZ area, perimeter, or circularity; however, a trend toward enlargement was observed in prediabetic patients. This may reflect the mild nature of retinal pathology in the prediabetic stage and the limited sensitivity of FAZ parameters in detecting very early disease.

Macular thickness measurements are widely used to assess diabetic macular edema. While some studies have shown thinner central macula in diabetics without retinopathy, others have reported similar thickness to healthy controls.^28^ In prediabetes, reductions in central thickness have also been reported.^29^^-^^31^ In this study, CMT did not differ between groups, revealing significantly greater thickness in the nasal and inferior regions of the outer retina and the inferior region of the inner retina in prediabetic patients. These findings can be interpreted as consistent with subclinical macular edema, likely reflecting early metabolic stress in the retina.

One important advantage of OCTA over FFA is its ability to separately image the SCP and DCP, where the earliest microvascular alterations occur. Histopathologic studies and OCTA analyses both suggest that the DCP is particularly vulnerable to early hyperglycemia-induced changes.^32^^,^ ^33^^,^ ^18^ Consistent with this, the findings of selective DCP involvement in prediabetes support the concept that retinal microvascular damage begins before overt diabetes or clinically detectable retinopathy.

### Limitations

First, the duration of prediabetes is uncertain. Individuals’ metabolic status may have changed over the 3-month period, which may have contributed to the variability in the results. Second, a larger sample size could have been provided, which would have strengthened the findings statistically and increased their generalizability. Third, although segmentation was manually corrected when necessary, OCTA remains susceptible to motion artifacts and image quality variability. Although the groups were age-matched, the gender distribution was not. Previous studies have reported gender differences in retinal microvasculature in patients with diabetes;^34^ this should be considered a limitation of the study. Finally, patients with neurological disorders such as Alzheimer’s disease, in which microvascular changes have been demonstrated in OCTA studies were not excluded;^10^ this potential confounding factor should be considered when interpreting the results, and its relationship with age should be considered accordingly.

In conclusion, the study demonstrates that prediabetic patients exhibit localized reductions in SCP and DCP PD and increased macular thickness in specific ETDRS regions, while FAZ parameters did not differ significantly from controls. These changes, though subtle, may represent the earliest retinal microvascular alterations in prediabetes. The OCTA, as a noninvasive and reproducible imaging tool, shows promise for detecting subclinical microangiopathy and may aid in identifying high-risk individuals for early intervention. Larger, well-characterized cohorts and long-term follow-up studies are essential to substantiate and generalize these results.

The findings indicate that retinal microvascular alterations begin in the prediabetic stage, even before the onset of clinically detectable retinopathy. Specifically, localized reductions in SCP and DCP vessel density and increased macular thickness were observed in selected ETDRS regions, while FAZ parameters remained unchanged. These subtle but measurable changes suggest that OCTA can serve as a sensitive, noninvasive biomarker for early detection of subclinical microangiopathy in prediabetes. Incorporating OCTA into screening protocols for prediabetic individuals may therefore facilitate timely identification of high-risk patients and open the door to preventive interventions before irreversible retinal damage occurs.

## Figures and Tables

**Table 1.** Comparison of Perfusion Density

Region/Parameter	Patients (n = 30), Mean ± SD (%)	Controls (n = 30), Mean ± SD (%)	*P*
DCP–Superior	17.13 ± 5.63	43.87 ± 8.50	.015
DCP–Nasal	17.60 ± 5.01	43.80 ± 6.90	.035
DCP–Inferior	16.00 ± 5.89	42.87 ± 8.05	**.005**
DCP–Temporal	16.07 ± 5.36	42.37 ± 6.78	**.005**
SCP–Superior	11.57 ± 3.98	31.60 ± 8.84	.053
SCP–Nasal	11.30 ± 3.36	35.83 ± 6.13	.047
SCP–Inferior	10.93 ± 2.50	36.60 ± 7.88	.039
SCP–Temporal	10.03 ± 4.08	35.33 ± 7.04	**.005**
SCP–Central	2.63 ± 4.48	3.90 ± 3.63	.234

Deep capillary plexus (DCP) and superficial capillary plexus (SCP) densities were significantly lower in patients, especially in inferior and temporal quadrants. Bold values indicate statistically significant differences (p < 0.05).

**Table 2.** Comparison of Foveal Avascular Zones

Parameter	Patients (n = 30) Mean ± SD	Controls (n = 30) Mean ± SD	*P*
FAZ area (mm^2^)	0.38 ± 0.22	0.31 ± 0.12	.122
FAZ perimeter (mm)	2.88 ± 1.08	2.60 ± 0.69	.238
Circularity	0.51 ± 0.09	0.33 ± 0.11	.159

Patients showed larger and less circular FAZ, but no statistically significant differences were found.

**Table 3.** Comparison of Macular Thickness and Ganglion Cell Layer Thickness

Region	Patients (n = 30) Mean ± SD (µm)	Controls (n = 30) Mean ± SD (µm)	*P*
ETDRS outer–Superior	327.1 ± 27.68	315.8 ± 16.94	.061
ETDRS outer–Nasal	337.0 ± 25.87	317.3 ± 20.40	**.002**
ETDRS outer–Inferior	331.0 ± 24.09	314.1 ± 14.92	**.002**
ETDRS outer–Temporal	321.0 ± 26.34	314.1 ± 13.78	.213
ETDRS inner–Superior	346.8 ± 30.10	322.3 ± 16.90	.059
ETDRS inner–Nasal	341.7 ± 27.66	317.2 ± 13.97	.012
ETDRS inner–Inferior	338.7 ± 32.38	317.5 ± 20.51	**.004**
ETDRS inner–Temporal	324.7 ± 27.81	313.8 ± 20.79	.090
ETDRS inner–Central	274.2 ± 39.12	264.0 ± 26.43	.245
GCC–Superior	113.6 ± 16.80	98.3 ± 9.06	.067
GCC–Inferior	110.8 ± 15.63	104.2 ± 9.00	.051

Patients had significantly thicker nasal and inferior macular regions, suggesting early subclinical retinal changes.
