# Erratum

**DOI:** 10.5152/eurasianjmed.2026.261702

**Published:** 2026-02-19

In the article by Utlu B, Utlu ES, Çinici E, Akgöz H, Bayrakçeken K, Kozan BD., entitled “Optical Coherence Tomography Angiography–Based Evaluation of Foveal Avascular Zone and Macular Vessel Density in Prediabetic Patients” that was published in the Volume 58 Issue 1 of The Eurasian Journal of Medicine (Eurasian J Med. 2026, 58 (1), 1165, doi:10.5152/eurasianjmed.2026.251165.), doi was mistakenly written as 10.5152/eurasianjmed.2026.261165.

The error was corrected on 06.01.2026 and the updated version of the article is available online at https://www.eajm.org/index.php/pub/article/view/3241

The corrected doi is: 10.5152/eurasianjmed.2026.251165.

